# Assessment of Genetic Diversity and Population Structure in Iranian Cannabis Germplasm

**DOI:** 10.1038/s41598-017-15816-5

**Published:** 2017-11-15

**Authors:** Aboozar Soorni, Reza Fatahi, David C. Haak, Seyed Alireza Salami, Aureliano Bombarely

**Affiliations:** 1 Department of Horticulture Sciences, Faculty of Agriculture, University of Tehran, Karaj, 31587 Iran; 2 Department of Horticulture, Virginia Tech, Blacksburg, VA 24061 USA; 3 Department of Plant Pathology, Physiology, and Weed Science, Virginia Tech, Blacksburg, VA 24061 USA

## Abstract

*Cannabis sativa* has a complex history reflected in both selection on naturally occurring compounds and historical trade routes among humans. Iran is a rich resource of natural populationswhich hold the promise to characterize historical patterns of population structure and genetic diversity within *Cannabis*. Recent advances in high-throughput DNA sequencing technologies have dramatically increased our ability to produce information to the point that it is now feasible to inexpensively obtain population level genotype information at a large scale. In the present investigation, we have explored the use of Genotyping-By-Sequencing (GBS) in Iranian cannabis. We genotyped 98 cannabis samples 36 from Iranian locations and 26 accessions from two germplasm collections. In total, 24,710 high-quality Single Nucleotide Polymorphisms (SNP) were identified. Clustering analysis by Principal Component Analysis (PCA) identified two genetic clusters among Iranian populations and fineSTRUCTURE analysis identified 19 populations with some geographic partitioning. We defined Iranian cannabis in two main groups using the results of the PCA and discovered some strong signal to define some locations as population according to fineSTRUCTURE analyses. However, single nucleotide variant analysis uncovered a relatively moderate level of variation among Iranian cannabis.

## Introduction

*Cannabis sativa* L. is a dioecious species in the Cannabaceae family^1^ with a broad global distribution which is likely the result of human cultivation. Humans have cultivated the plant as a source of fiber, food, medicines, intoxicants and oils for thousands of years^1,2^. This use and breeding has led to the selection of two distinct types of *C. sativa*, one for fibre and seed (hemp) and one for medicinal use (marijuana). While these types are morphologically similar, they are distinguished by the type and level of cannabinoids produced. Levels of two types of cannabinoids in particular are used to distinguish marijuana and hemp *C. sativa*. First, D-9-tetrahydrocannabinol (THC) is a psychoactive compound^3^ found in leaves and inflorescences (but not seeds) of juvenile and mature plants. The second compound, cannabidiol (CBD), is an isomer of THC found in all plant tissues, however, this cannabinoid does not activate cannabinoid receptors^1,4,5^. Marijuana varieties used for drug consumption are characterized by a high THC content, whereas fibre varieties (hemp) produce CBD as the predominant cannabinoid^6,7^.

Archaeological and palaeobotanical evidence supports the cultivation and use of *Cannabis* since the Neolithic period with subsequent secondary domestication events in geographical regions outside of the accepted native range^8–15^. For instance, archaeological evidence for the pharmaceutical or shamanistic use of *Cannabis* has been found in cave artifacts that include a large cache of *Cannabis* dating to ca. 700 BCE^16^. This long history of use has resulted in a complex biogeographical history for this species. Based on polymorphism in RAPD markers, the Eurasian Steppe region of Central Asia has been recognized as a putative center of origin for *Cannabis*, spreading from there to the Mediterranean as well as Eastern and Central European countries, in particular, Afghanistan and Pakistan^17^. However, the genus has also been described has having two centers of diversity, Hindustani and European–Siberian^18^. As with other cultivated plants it is difficult to pinpoint the exact place of origin for *C. sativa*. It is likely that *Cannabis* spread to ancient Persia very early, assisted by Aryan and Scythian tribes expanding westward from central Asia. Evidence for this early spread comes from archeological studies of the Scythians, who occupied an area encompassing large swathes of what is now northwest Iran from the 7th century BCE to the 4th century CE, this culture was known to use *Cannabis* for entertainment and spiritual purposes. While all Iranian cannabis has been described as a complex of landraces of *C. sativa*, it is one of the countries with a high level of genetic diversity among cannabis populations^1,19^.

Currently, the most important topics in *C. sativa* genomics and transcriptomics are, (1) Identification of sex determining regions^20–23^, (2) forensic investigations^24,25^, (3) selection of the chemotype and identification of hemp and marijuana types^13,26^, (4) DNA typing and genetic relatedness analyses^27^, and (5) the development of molecular markers for distinguishing hemp and marijuana genotypes as well as determining genetic structure and diversification through domestication^28–30^.

A draft genome and accompanying transcriptome of *C. sativa* were published in 2011^31^ and a large scale study of the genetic structure of marijuana and hemp types was published in 2015^30^. Nevertheless, the phylogeography and domestication history of *Cannabis* remains poorly understood, in part due to limited access to genetic material from natural populations. Given that *Cannabis* is a native plant with a long history of cultural use in Iran, it is surprising that no studies of *Cannabis* diversity using molecular markers exist. Here we present an initial description of population structure and genetic diversity, between Iranian and global collections of *Cannabis* as well as within the Iranian collection. Specifically, we leverage genotyping-by-sequencing (GBS)^32^ to generate single nucleotide polymorphisms (SNPs) across a large collection of Iranian cannabis. GBS provides a robust, cost-effective alternative to other approaches and provide greater power to detect genome wide patterns associated with population structure and demographics than other molecular markers^33,34^.

## Results

### Sequencing and mapping

**Figure 1:**
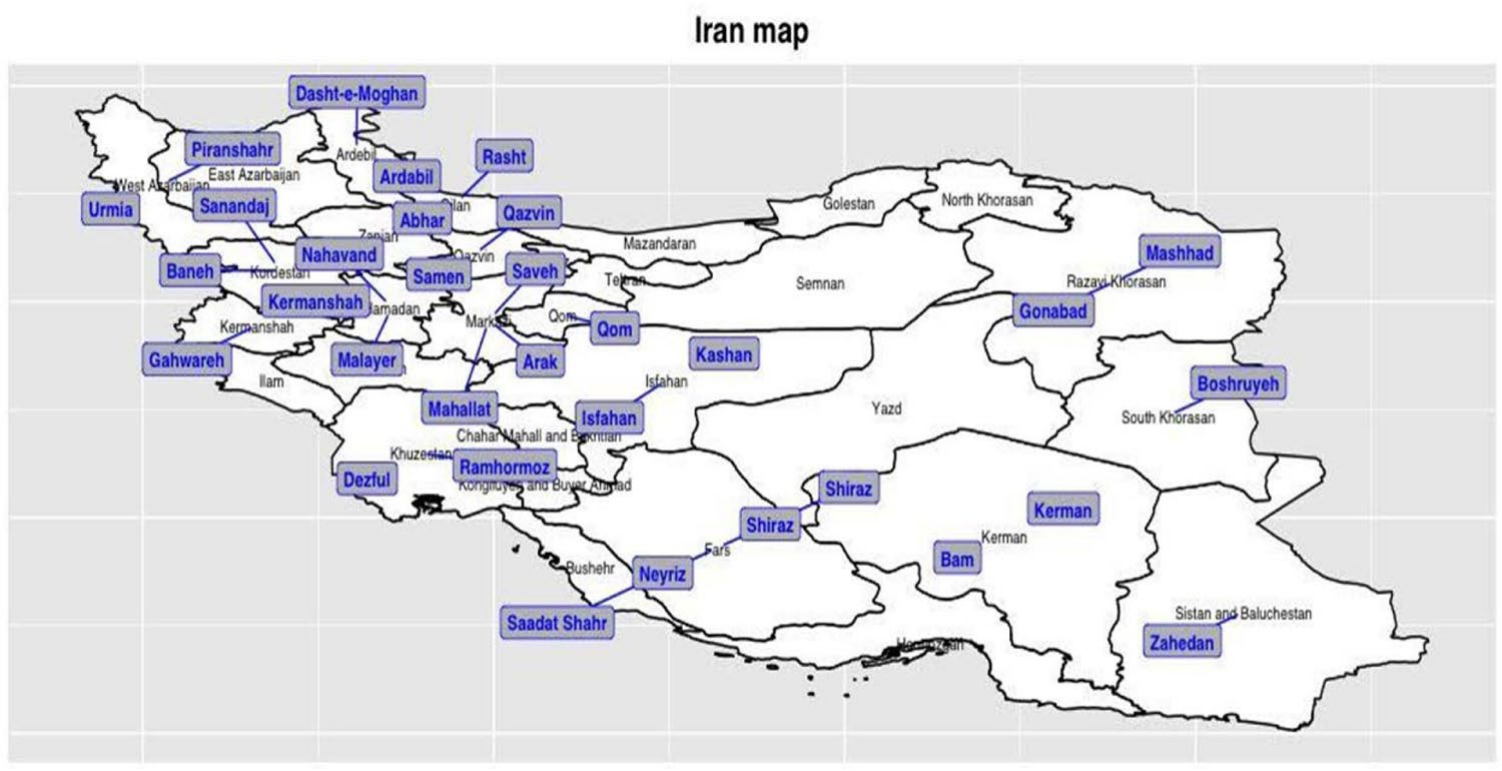
Geographical distribution of samples across Iran. This figure was produced using the R software version 3.1.3 with the packages: Raster version 2.5–8 (https://cran.r-project.org/web/packages/raster/index.html) and Ggplot2 version 2.2.1 (http://ggplot2.tidyverse.org/reference/)^59^.

In total 98 cannabis samples were digested, sequenced, and genotyped these included, 70 samples representing 35 locations in Iran (Fig. 1), 2 samples from Afghanistan and 26 accessions provided by CGN (Center for Genetic Resources, The Netherlands) and IPK (The Leibniz Institute of Plant Genetics and Crop Plant Research, Germany). For each location or accession one female and male plant was sampled. After quality filtering a total 431.3 million raw sequence reads were obtained from 100 bp single-end sequencing on the Illumina HiSeq. 2500 Rapid Run platform. Three individual samples representing three different locations (Afg-M, Esf-M and Kash-F) were removed because they had fewer than 1 M reads each. The remaining samples were represented by a mean of 4.4 M reads (range 1.58 to 14.53 M) per individual (Table S1). Approximately, 89% and 83% of the GBS reads mapped to the *C. sativa* Purple Krush and Finola reference genomes, respectively. These uniquely mapped sequence reads covered approximately 0.43% to 1.08% of Purple Kush genome (estimated size ~786.6 Mb). Because the Finola genome assembly is incomplete (~221.4 Mb), the percentage of genome representation was higher, from 1.26% to 3.08%.

**Figure 2:**
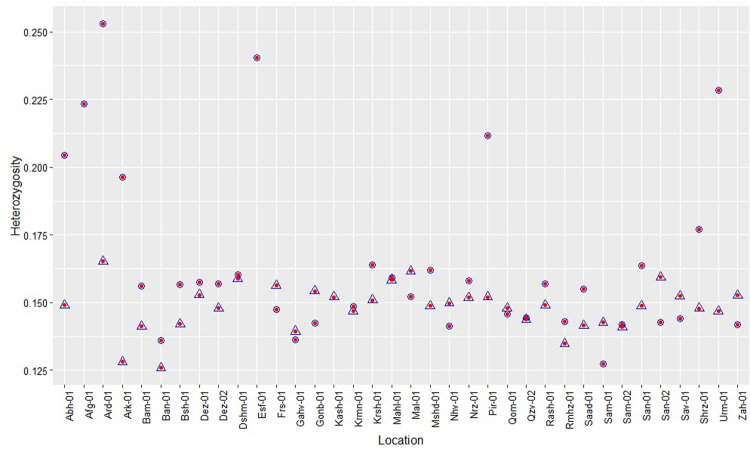
Heterozygosity per location. Triangles represent male samples and circles represent female samples.

Sample level variation in the percentage of reads mapped to the reference (Table S1) revealed individuals with a mapping percentage below 70%, specifically; 883049-M with highest read rate (14.54 million), 901072-M, CAN18-M, CAN47-M, 901072-F and CAN57-F, all from the germplasm collections of CGN and IPK. These differences may arise from differences in sequencing depth across regions, excessive amplification in the PCR step, short read length, or problems with the sequencing platform^35^. The number of markers ranged from 2.7 to 8.5 million with average distance of 5089 bp and 2.4 to 6.8 million with distance of 780 bp on Purple Kush and Finola genome, respectively.

### SNP Discovery, Heterozygosity and Genetic Differentiation

After quality filtering, 24,710 high-quality SNPs were identified across all samples and 29,647 SNPs were identified for 68 Iranian individuals, including one Afghanistan sample. This set of filtered SNPs were used for subsequent analysis, and had average distances of 4241 and 621 bp on Purple Kush and Finola genomes repectively. The transition:transversion ratio was 1.65 (Fig. S1). The majority of SNPs (62.7%) detected were transitions (A/G or C/T) while transversion events (A/C, A/T, C/G or G/T) accounted for 37.3%. The ratio of transitions to transversions is consistent with other studies in various species^36–39^. Tajima’s D was calculated for the filtered SNPs with a mean of −0.18 (range, −2.16 to 3.70) across all samples (Fig. S2) and a mean of 0.007 (range, −1.95 to 3.55) for the 68 samples originally from Iran. Tajima’s D is a summary statistic often used for identifying selective sweeps from genomic data, where values are 0 for neutral variation, positive when an excess of rare polymorphism indicates positive selection, and negative with an excess of high-frequency variants, which indicates balancing selection^40^. The distribution of Tajima’s D among Iranian cannabis samples suggests that balancing selection likely shaped genetic structure across these populations (Fig. S2). This pattern is common among groups that experience heterozygote advantage, wherein rare alleles are retained at low frequencies. Average heterozygosity was estimated at 0.15 across 68 samples originally from Iran and an Afghanistan. This estimate of heterozygosity is similar to that found by Sawler *et al*.^30^ for marijuana type accessions. Samples Ard-01-F and Esf-01-F from Ardabil and Isfahan states showed the highest number of heterozygous sites (Fig. 2, Table S1).

Population differentiation resulting from genetic structure was estimated using F_ST_. For the Iranian samples, the minimum F_ST_ was −0.058, calculated between Saad and Esf locations, and the maximum F_ST_ was 0.26 for Gahv vs Ard, locations that are separated by 434 km (Fig. 1, Table S2). Low values indicate that genetic diversity is higher within individuals from these locations than between locations, a pattern consistent with gene flow between populations. F_ST_ estimates above 0 indicate a reduction in genetic exchange between population with a value of 1 indicating complete isolation. Across all individuals the maximum F_ST_, 0.425, was estimated between non-Iranian samples 883049_vs_CAN37. Sample 883049 (from kompolti Sargâszâru) has been identified as a fiber cultivar^41^. CAN37 was previously described as hemp type and originating in France, however, Sawler *et al*.^30^ found that it was a distinct outlier and was more closely associated with marijuana and speculated that it could be a mislabeled sample. We also estimated genetic differentiation among marijuana and hemp accessions and Iranian samples and found a larger F_ST_ across hemp 0.086 than marijuana 0.039.

**Figure 3:**
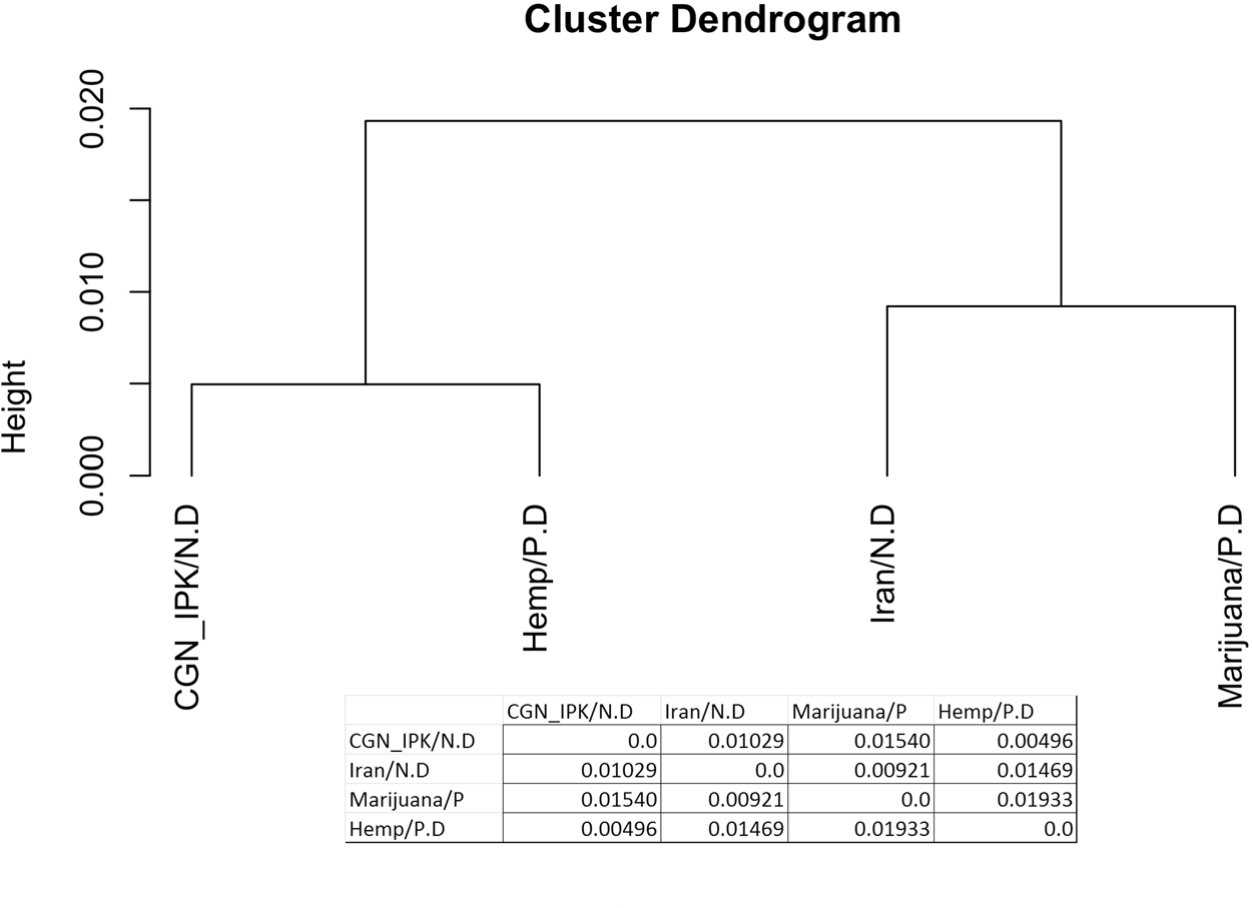
The dendrogram generated from Nei’s genetic distance.

Nei’s genetic distance^42^ was evaluated on 13,325 SNPs that were identified across 209 samples (all data, including that from Sawler *et al*.^30^) as another metric of genetic relationships among types and collections. Nei’s genetic distance values ranged from 0.00496 to 0.01932 and largely reflected the DAPC analysis. Similar to Sawler *et al*.^30^, hemp showed the least genetic distance followed by germplasm collections from CGN and IPK. Marijuana and Iranian cannabis clustered together with genetic distances of 0.00496 and 0.00921, respectively, while the genetic distance between Iranian collections and hemp was estimated at 0.01469 (Fig. 3). Overall, these results suggest that Iranian collections are more genetically similar to marijuana collections than hemp.

### Gender, Drug and Non-Drug

To identify of DNA markers associated with gender for rapid/early identification of male and female plants, we examined allele frequency differences between female and male samples at the same position, in a modified bulked segregant analysis. It is important to note that neither of the reference genomes used in this study were from a male plant. Our approach failed to identify sex specific alleles at high frequency outside of the sex determining region.

Previous analyses have shown that marijuana and fibre types differ across the genome and not just at specific loci. Our approach failed to identify positions with significant deviations in allele frequency among 19,345 SNPs between types. Sawler *et al*.^30^ reported a highest allele frequency of 0.82 in hemp and 0 in marijuana for a single polymorphism. Our reanalysis of these data identified 9 SNPs with allele frequencies of 1 for hemp and 0 for marijuana and 92 SNPs with allele frequency 0 for hemp and 1 for marijuana. All positions and their frequencies are supplied in Table S3.

### Population structure

**Figure 4:**
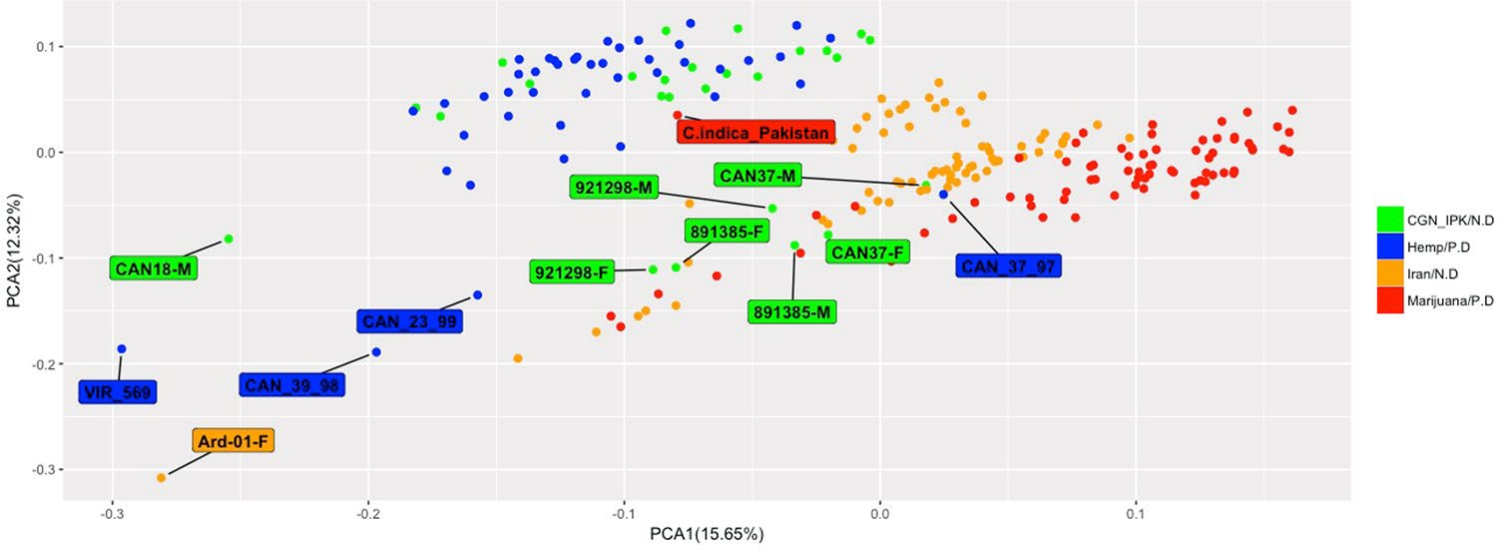
Principle components analysis of 95 samples from Iranian collection, 43 hemp and 71 marijuana samples using 13,325 SNPs. Hemp samples are colored blue and marijuana samples are colored red. Iranian samples divided to two groups, Original from Iran (orange) and come from germplasm collections of CGN and IPK (green) (N.D stand for New Data and P.D stand for previously analyzed data).

**Figure 5:**
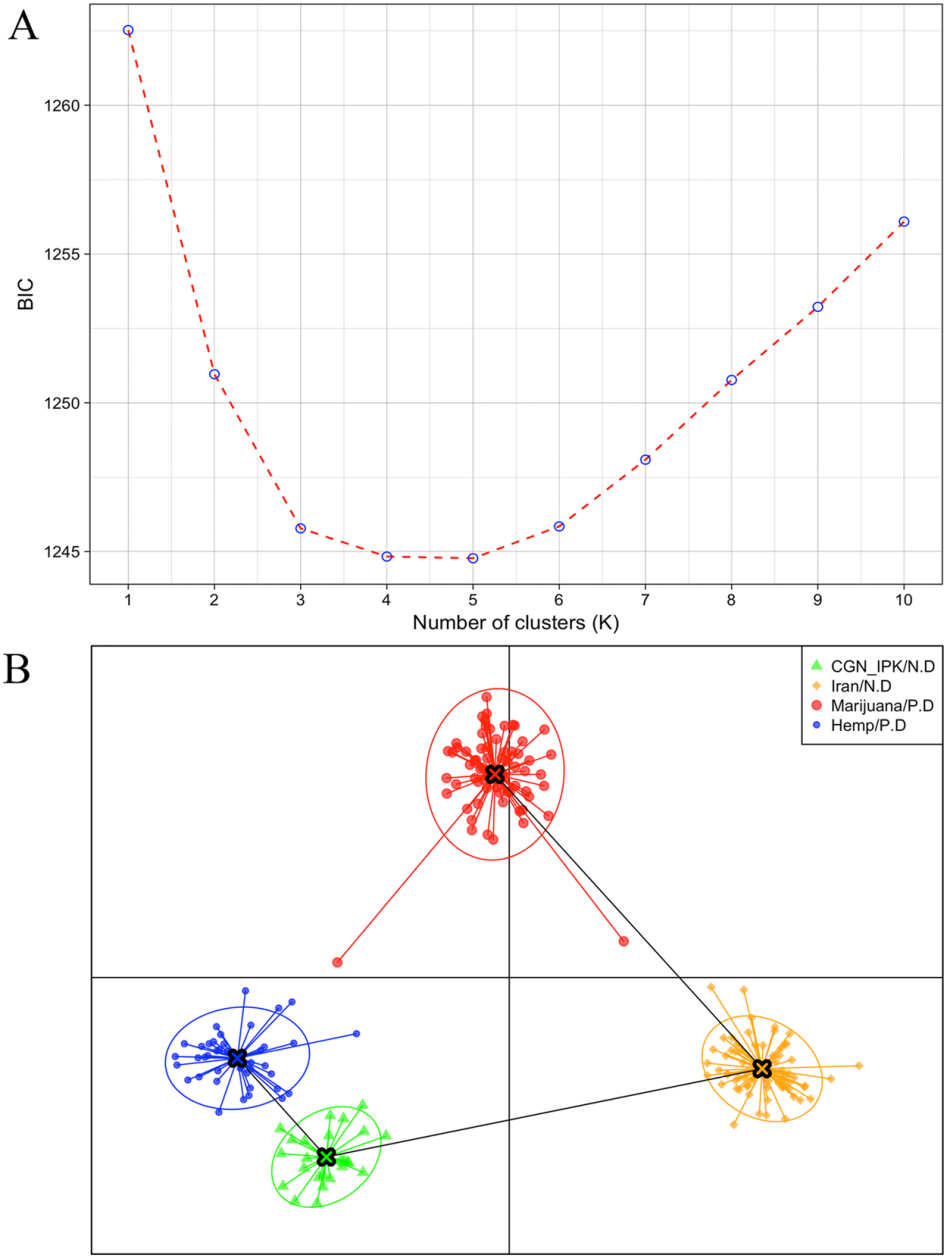
Discriminant analysis of principal components (DAPC) results. (**A**) The optimal number of clusters (K) as determined by ‘k-means’. The graph shows a clear decrease of BIC until k = 4 clusters to be the most likely value of K, after which BIC increases. (**B**) Scatterplot based on the DAPC output for four assigned genetic clusters, each indicated by different colours. Dots represent different individuals.

An initial analysis of population structure was performed using individual-based principal component analysis (PCA). PCA using data from Iranian collections, CGN (A fiber germplasm collection execpt for one accession, 891385 which known as a drug cannabis)^43^ and IPK (A hemp germplasm collection), and *C. sativa* GBS data from Sawler *et al*.^30^ (Fig. 4) revealed two main clusters supporting the reported split between marijuana and hemp accessions^30^ and revealed that Iranian collections tend to cluster with marijuana accessions. This plot revealed two nonconforming individuals (CAN18-M and Ard-01-F) that failed to group with the two main clusters. Previous outliers from Sawler *et al*.^30^ were suggested to be sample error or misclassification (hemp vs. marijuana), our data suggests that CAN individuals (CAN37 from our collection and CAN23_99, CAN39_98 and CAN_37/97 from Sawler *et al*.)^30^ are more genetically similar to marijuana type accessions. To further elucidate genetic clustering identified by PCA, we performed a Discriminant Analysis of Principal Components (DAPC)^44^. Consistent with fineSTRUCTURE analysis (Fig. S5), DAPC identified 4 distinct clusters (Fig. 5A). Visualisation of DAPC results using the first 22 principal components clearly clusters, marijuana, hemp, germplasm collections, and Iranian collections (Fig. 5B).

**Figure 6:**
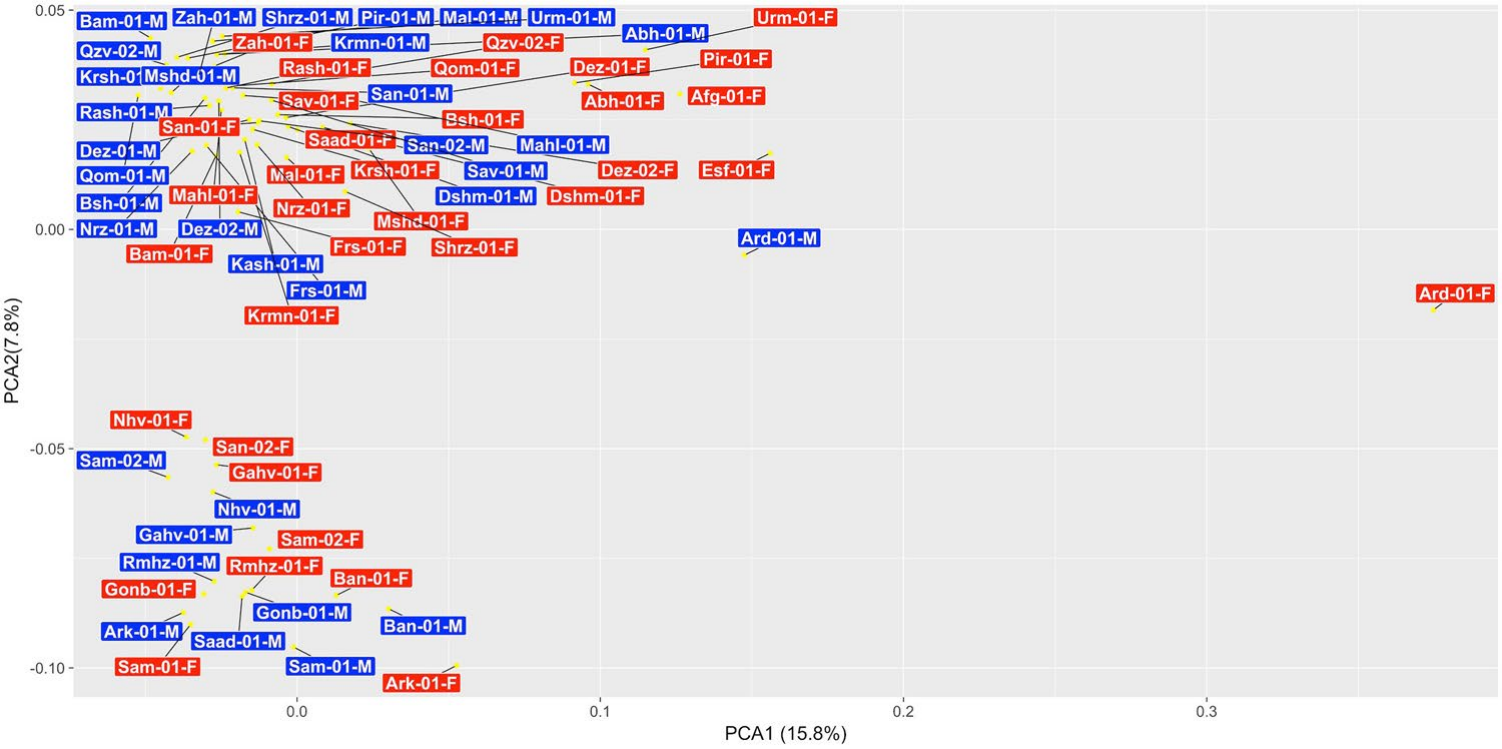
Individual-based principal components analysis for 35 Iranian regions and Afghanistan using 29,647 SNPs. Male plants are colored blue and female plants are colored red.

PCA within the Iranian collection identified two primary clusters (Fig. 6) separated along principal component 2, representing 7.8% of variance. This clustering separated accessions from Sanandaj, Samen, Ramhormoz, Gahwareh, Gonabad, Baneh, Arak and Saadat Shahr (Iran’s western margin states) and the rest of Iranian accessions. These inferences were also largely consistent with results from a fastSTRUCTURE analysis. Notably, fastSTRUCTURE identifies 2 genetic clusters within Iranian cannabis (Fig. S4). Gene flow estimates between these clusters, identified via MIGRATE-N^45,46^, indicates an asymmetric sharing of alleles (Table S4) between clusters. This pattern is consistent with reduced gene flow from cluster 1 which includes 18 samples (Fig. 6) such as Rmhz, Gonb, Gahv, Ark, Sam, Nhv, San-01, Ban and Saad-01-M and cluster 2 with all other samples. Genetic clustering with fineSTRUCTURE identified genetic structure among individuals from the same region (Fig. S4, Table S5). According to these results we can define distinct genetic clusters for locations Neyriz, Piranshahr, Gahwareh, Arak, Urmia and Abhar. Analysis of Molecular Variance (AMOVA), as implemented in Arlequin^47^, on the two and 19 genetic clusters obtained by fastSTRUCTURE and fineSTRUCTURE respectively, found that variation within populations was very high (93.09% and 95.74%) compared to between population estimates (1.38% and 1.02%; Table S6). This pattern is consistent with perennial dioecious plants wherein the majority of variation is harbored within populations^48^. Together these suggest that Iranian cannabis populations tend to share more DNA with geographically proximate populations where may have genomes made up of mixtures of inferred source populations, while our simulation incorporated drift between locations, but not admixture.

## Discussion

*Cannabis*, both marijuana and fibre types, is a globally important plant, driving a multi-billion dollar industry. Unraveling the population genomic parameters of natural populations can help identify sources of genetic diversity, as well as describing patterns of domestication for this widely used plant. In this study, we have found that natural populations of *Cannabis* in Iran are more closely related to marijuana than hemp, and that these populations harbor unique pools of genetic diversity. Taken together these data support the hypothesis that reduced diversity across fibre types suggests that hemp cultivars are derived from marijuana^30^.

Population analyses among all accessions sampled defined 4 distinct genetic clusters (Figs 3,4 and 5). These analyses support previous findings (Sawler *et al*.^30^) that marijuana and hemp are differentiated and identify Iranian collections as genetically more similar, yet distinct from, marijuana. This evidence provides support for the hypothesis that Iranian cannabis harbors unique genetic diversity and may represent a distinct genetic lineage of marijuana. Heterozygosity indicates levels of genetic diversity within populations, and has also been used to estimate genetic distance between populations^49,50^. Consistent with genetic diversity levels in the present study, previous estimates of heterozygosity across diverse marker types (e.g., SNP, SSR, AFLP) typically identify higher levels of heterozygosity in hemp compared to marijuana^30,51–53^. However, it should be noted that one study found lower levels of heterozygosity in hemp varieties across 195 samples and 2894 SNPs^29^. It has been suggested that this may result from limited hemp sample representation in the collection^29^. Heterozygosity estimates within our Iranian collection were similar to those found by Sawler *et al*.^30^ for marijuana type accessions. If, as we surmise, Iranian cannabis are marijuana accessions, then these accessions likely represent remnants of cultivated germplasm from the other regions, possibly through migration of *Cannabis* from neighboring countries like Afghanistan and Pakistan into Iran. These results demonstrate that Iran is a public repository of marijuana genetic diversity; however, the loss of this unique germplasm is of great concern as there are no breeding programs and growing *Cannabis* is associated with strict legal penalties.

PCA and fastSTRUCTURE analysis of the Iranian collection identified two genetic clusters (Fig. 6, S4) separated along an east west gradient. Further analyses via fineSTRUCTURE showed that some locations are supported as distinct genetic populations (Fig. S3). These observations reveal that Iranian cannabis, despite clear evidence of admixture (likely the result of breeding), harbors distinguishable pools of genetic diversity. The lack of strong population differentiation is unsurprising since, all known cultivars of *Cannabis* are wind-pollinated and highly heterozygous (confirmed by AMOVA, Table S6). Population structure is further complicated by the fact that marijuana cultivars are clonally propagated in order to retain high-levels of THC production. Intentionally growing *Cannabis* plants in Iran is punishable by prison sentence, populations of plants are more likely to have arisen from seed and therefore represent more natural populations. Although Iranian cannabis is not likely a subspecies it does represent a genetically unique variety of marijuana, and thus provides a novel source of genetic material for cultivar development.

In plants, the sex determination system is important for two reasons; first, understanding the role of sex determination in shaping plant evolution, and second, diversity in the mechanisms through which sex is determined. There have been many studies on gender in *Cannabis*, including whether a plant should be classified as female or male, and in addition to the identification of sex chromosomes^21^, some male-specific DNA markers have been identified in *C. sativa*, allowing verification of gender during early developmental stages^20,22,54^. Sex determination in *Cannabis* is a complex process and can be modified or reversed by environmental factors and chemical treatment^55,56^. Additionally, male flowers are able to develop on female plants under extreme conditions^57^. Because confirmed sex-associated DNA markers such as MADC2 sometimes fail to discriminate sex phenotype^22^, we attempted to identify sex associated markers from autosomal regions. While our study generated thousands of differentiating markers, we failed to find sex locus specific SNPs. This is likely because no male reference genome is available and the proportion of coding regions covered by the GBS derived SNPs. Future studies can capitalize on the utility of high-throughput sequencing technologies to look for markers associated with sex-determining loci, in particular coding derived SNPs (e.g., RNA-seq). We were able, however, to identify marijuana and fibre type specific markers through reanalysis of previously published data.

Our conclusions, consistent with previous studies, show that genetic differences between hemp and marijuana accessions are widely distributed across the genome^30^. Comparative analysis of Purple Kush (marijuana) and Finola (fibre) genomes revealed highly discriminative SNPs that are distributed across the genome and are not restricted to particular loci (e.g., cannabinoid production)^31^. While previous work focused on THC:CBD ratios and the associated B locus (a single locus with two co-dominant alleles)^41^, recent work has identified SNPs in THCA and CBDA synthases associated with chemotype variation^58^. Thus, associating SNPs with active and inactive forms of THC and CBD synthases will continue to be a powerful tool for distinguishing *Cannabis* types. In this study, we identified SNPs that appear to be tightly linked to type, and are outside of cannabinoid genes, which should prove useful for future research. More immediately, these markers can be validated for early and rapid identification of marijuana and fibre type plants for current breeding programs.

## Materials and Methods

### Collection of Genetic Material

Natural populations of *Cannabis* in Iran were identified and seeds were collected for growing in the field in university of Tehran. Sex identities were verified using taxonomic keys. A set of different accessions provided by CGN and IPK and one population from Afghanistan were used for analysis in this study as well (Table S1, Fig. 1). Figure 1 was produced using the R software version 3.1.3 with the packages: Raster version 2.5-8 (https://cran.r-project.org/web/packages/raster/index.html) and Ggplot2 version 2.2.1 (http://ggplot2.tidyverse.org/reference/)^59^. Additionally Dplyr version 0.5.0 was used to manipulate the dataframes (https://cran.r-project.org/web/packages/dplyr/index.html).

### DNA Extraction, Library Preparation and Sequencing

DNA was extracted using a Qiagen DNeasy plant mini-kit, from leaf tissue of one female and one male plant from each location. The isolation procedure was carried out according to the manufacturer’s’ guidelines. We performed *in silico* digestion of the Cannabis genome sequence with *Pst*I and *Apek*I to select the best restriction enzyme library preparation. Libraries were prepared using the GBS protocol published by Sonah *et al*. (2013). A 150 ng genomic DNA template was used to prepare the library using the *Apek*I enzyme. High-throughput was performed on an Illumina Hiseq. 2500, Rapid-run mode, single-end 100 base reads, at Duke Center for Genomics and Computational Biology.

### Bioinformatics Analysis

#### Demultiplexing and Read Filtering

After unzipping fastq.gz files to fastq files by gunzip command, the GBSX package^60^ was used for demultiplexing of reads. Reads were organized into new files with adapter sequences removed, reads were discarded that were, shorter than 50 bases, and trim leading and trailing low quality regions (<Q30) by fastq-mcf, a widely available open source software^61^. To elucidate the relationship of Iranian cannabis with marijuana and fibre type accessions, we merged our data with marijuana and hemp data prepared by Sawler *et al*.^30^ (downloaded from NCBI SRA BioProject: PRJNA285813).

### Mapping, SNPs Discovery and filtering

In a high-throughput genotyping workflow, alignment of short reads to a reference genome is the first step after read processing and filtering. BWA^62^ was used to map reads of the individual genotypes to the reference genome with the default parameters. Reads mapped to Purple Kush (canSat3: a special variety of hemp) and Finola (finola1: a special variety of marijuana) *C. sativa* reference genome assembly separately which are known as high and low-THCA producing varieties respectively. The mapping outputs were used for removing unmapped reads to produce BAM files using Samtools^63^ and only reads mapping to a unique location in the genome were retained. Merging all BAM files into one stream by bamaddrg utility (https://github.com/ekg/bamaddrg), sorting and indexing BAM files by Samtools package^63^ were primary stages for use of FreeBayes^64^ to detect variants. Before running FreeBayes, we estimated the number of markers for each individual by “bedtools genomecov”^65^ and percentage of coverage by dividing marker number times read length by genome size. FreeBayes was run using default parameters. This was performed for or males and females and drug and non-drug types separately to find positions linked to gender and type. Bi-allelic, missingness, quality, and depth were filtered. The aim of the QC on SNPs was to define high quality set of individuals for analysis. Bi-allelic markers were identified by a command-line written in our lab. Then got vcflib freely available (https://github.com/vcflib/vcflib.git) packages to filter down the SNPs that had mapping quality <30 and read depth <5. This package can filter each position for each individual. Filtering was initially performed using VCFtools package^66^, VarFilter from BCFtools is freely available (https://github.com/samtools/BCFtools) packages. After screening a few markers we found that read depth and quality were not being appropriately filtered for our data set and therefore we opted to use vcflib. To filter missing data we used “–max-missing 1.0” option in VCFtools package^6^. Finally, summary statistics were collected using vcf-stats before and after data filtering.

### Scan for Identification of SNPs associated with gender and type

Identification of DNA markers associated with gender and type was carried out based on comparison of SNP allele frequency differences between each group (female-male and marijuana-fibre). To do this, we called SNPs for sample pairs female and male, marijuana and fibre, separately using FreeBayes^64^. After filtering variants for read depth (>5), read mapping quality (>30) and minor allele frequency (>2.5%), we generated allele frequency estimates and compared frequencies at the same position across the genome.

### Analysis of population structure

We computed the fixation index (F_ST_) using VCFtools^66^ among all wise locations in the Iranian collection and also between marijuana and hemp types. Estimation of heterozygosity for each individual was conducted with custom command-line scripts by dividing the number of heterozygous sites by the number of non-missing genotypes. The number of heterozygous sites was counted by vcflib tools. We pursued principal components analysis (PCA) to investigate genetic relationships using a distance matrix obtained by TASSEL version 5^67^. Plotting PCA results was completed via the ggplot2^59^ package in Rstudio version 0.99.902. We also applied discriminant analysis (DA) of principal components^44^ using the adegenet package^68^. Discriminant analysis can ascribe relationships for pre-defined groups without relying on a particular population genetics mode^44^. Files were read using the function read.vcf and converted into geneid objects with the vcfR2geneid function^69^. In DAPC, data is first transformed using a principal components analysis (PCA) and subsequently the number of genetic clusters was assessed using the find.clusters function. The Bayesian Information Criterion (BIC) was calculated for K = 1–10. For k-means clustering, all of the principal components were retained. The K value with the lowest BIC was selected as the optimal number of clusters. DAPC was implemented using the optimized number of principal components as determined by the optim.a.score function. Nei’s genetic distance^42^ among populations was calculated using the StAPP package for R^70^ and the resultant dendrogram was drawn using the standard R function plot.hclust. To determine the most probable number of genetic clusters, fastSTRUCTURE^71^ was run at K = 1 and K = 10, with an average of 22600 iterations, using default parameters for the Iranian samples. The analysis at K = 2 was performed to test the extent to which the samples reflect two distinct groups. Other values of K were tested (not shown), but did not provide further optimization or descriptive value. Additionally, the cannabis population structure was investigated using fineSTRUCTURE^72^. To visualize populations, we plotted the output data via the fineSTRUCTURE graphical user interface.

The genetic clusters from fastSTRUCTURE and fineSTRUCTURE were used to estimate gene flow and population size via MIGRATE-N (v. 3.6.11)^45,46^. In this case, gene flow was estimated between two clusters obtained by fastSTRUCTURE only for Iranian cannabis (69 samples). MIGRATE-N was implemented with following parameters: the Bayesian inference strategy, 1000 for number of recorded steps in chain, a burn-in of 1000 for each chain and a full migration model with two population sizes and two migration rates. The starting values for θ and M were generated initially from Fst, Migrate-n was subsequently run using the resulting θ and M values of the previous run. The runs were conducted on 5 K of markers. Hierarchical analysis of molecular variance (AMOVA) was performed using the Arlequin software package (v. 3.1)^47^. Significance levels for variance components and *F-statistics* were estimated using 1000 permutations.

## Electronic supplementary material

Supplementary Information

Supplementary Table S1

Supplementary Table S2

Supplementary Table S3
