# Supplementary material for: Assessment of Genetic Diversity and Population Structure in Iranian Cannabis Germplasm
Source: Sci Rep. 2017 Nov 15;7:15668. doi: 10.1038/s41598-017-15816-5 (PMC5688169; doi:10.1038/s41598-017-15816-5)
Supplement: Supplementary file 1 — Supplementary Information [file 41598_2017_15816_MOESM1_ESM.pdf]

Assessment of Genetic Diversity and Population Structure in Iranian Cannabis Germplasm

Supplementary Tables 4, 5 and 6

Supplementary Figures S1, S2, S3, S4 and S5

Aboozar Soorni[1,2], Mohammad Reza Fatahi[1], David C. Haak[3], Seyed Alireza Salami[1*], Aureliano Bombarely[2*]

[1]Department of Horticulture Sciences, Faculty of Agriculture, University of Tehran, Karaj 31587, Iran

[2]Department of Horticulture, Virginia Tech, Blacksburg, VA 24061, U.S.A.

[3]Department of Plant Pathology, Physiology, and Weed Science, Virginia Tech, Blacksburg, VA 24061, U.S.A.

*To whom correspondence should be addressed.

Authors E-mail

Aboozar Soorni:aboozar.soorni@gmail.com

Mohammad Reza Fattahi: fattahi@ut.ac.ir

David Haak: dhaak@vt.edu

Seyed Alireza Salami: asalami@ut.ac.ir

Aureliano Bombarely: aurebg@vt.edu

All loci	Mean	0.025	0.975	Nm
θ1	0.01289	0.0112	0.01453	
θ2	0.0053	0.0036	0.00693	
M2->1	25	8	41.3	0.08056
M1->2	999	948.7	982	1.32367

Table S4: Migration rates

Populations	Individuals
pop2	ArdF,ArdM
pop3	PirF,PirM
pop6	NhvF,SanF
pop7	NrzF,NrzM
pop8	GahvF,GahvM
pop10	UrmF,UrmM
pop11	RmhzM,GonbF,RmhzF,SaadM
pop12	NhvM,Sam02F,Sam02M
pop13	ArkF,ArkM
pop16	KashM,DezM,DshmM,KrshM,BshM,FrsM,Dez02M, MshdM,SanM,SavM,QomM,RashM, San02M
pop17	AbhF,AbhM
pop18	BamM,KrmnM,MalM,ZahM,ShrzM,Qzv02M
pop19	FrsF,DezF,BshF,BamF,DshmF,KrmnF,MalF,MshdF,SaadF, KrshF,MahlF,SanF,Dez02F
pop20	QomF,AfgF,EsfF,Qzv02F,SavF,ZahF,RashF

Table S5: clustering of samples into populations by fineSTRUCTURE

Groups	Source of variation	df	Variance components	Percentage of variation	*F-statistics*
fastSTRUCTURE (2 Clusters)	Among groups	1	146.84807	5.53	F_{CT}= 0.05525**
	Among populations within groups	31	36.73616	1.38	F_{SC}= 0.01463**
	Within populations	105	2474.11931	93.09	F_{ST}= 0.06908**
FineSTRUCTURE (19 Clusters)	Among groups	18	137.71115	3.24	F_{CT}= 0.05283**
	Among populations within groups	14	26.63795	1.02	F_{SC}= 0.01079*
	Within populations	105	2495.5299	95.74	F_{ST}= 0.04261**

Table S6: Analysis of molecular variance (AMOVA) for 29,647 SNPs identified for 68 Iranian individuals, including one Afghanistan sample.

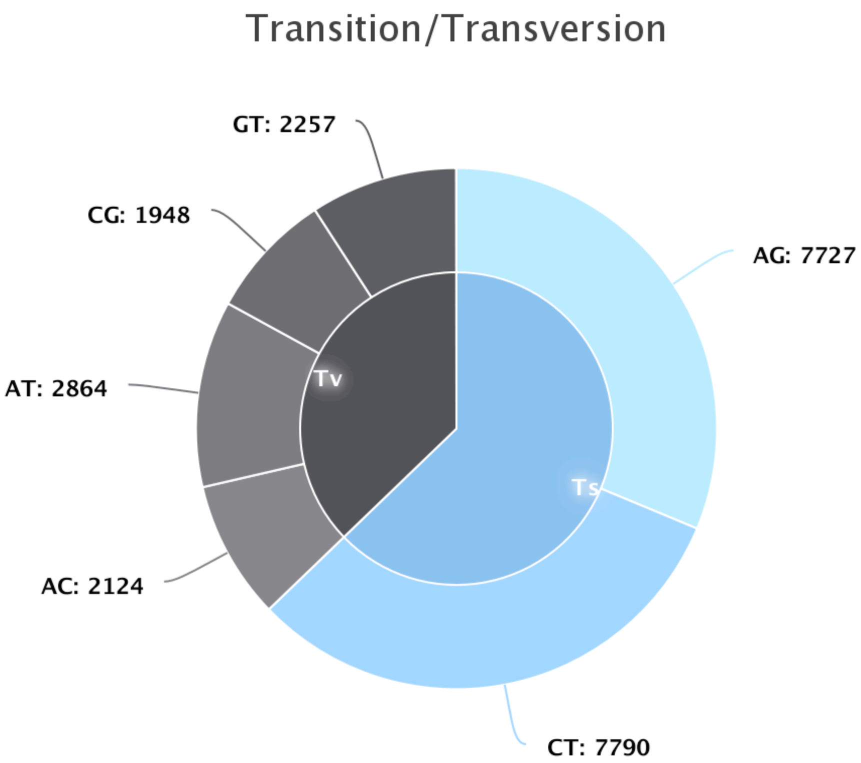

Figure S1: Estimation of the Transition/Transversion Rate

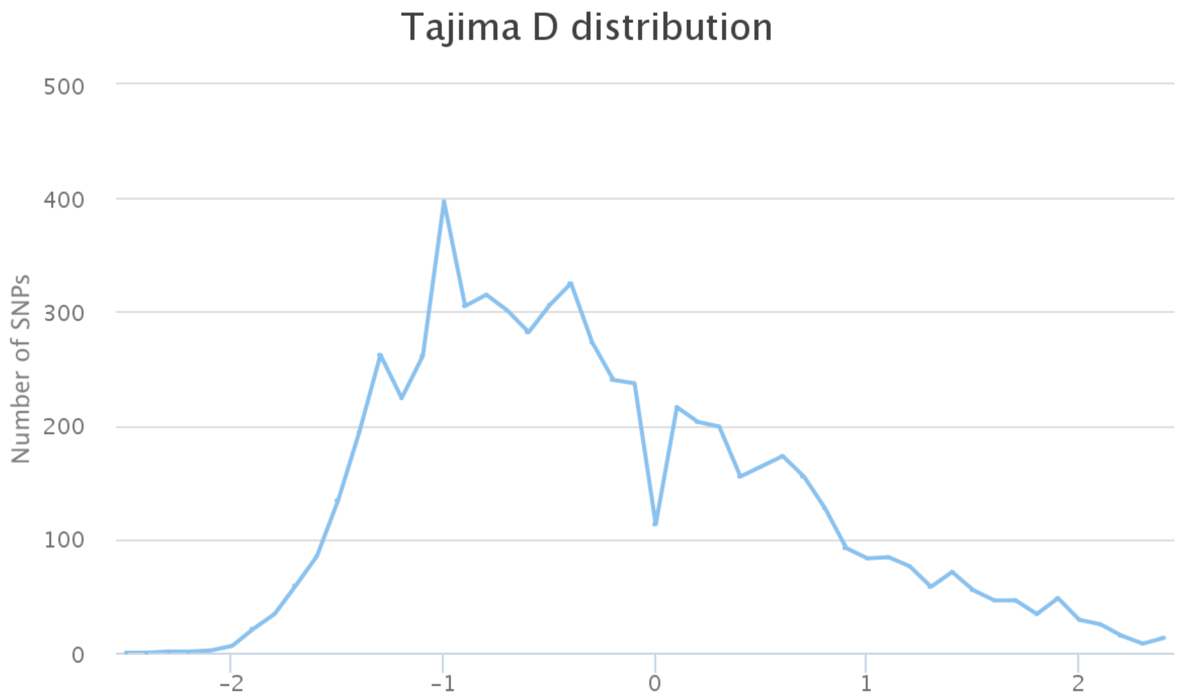

Figure S2: Distribution of Tajima's D value along SNPs

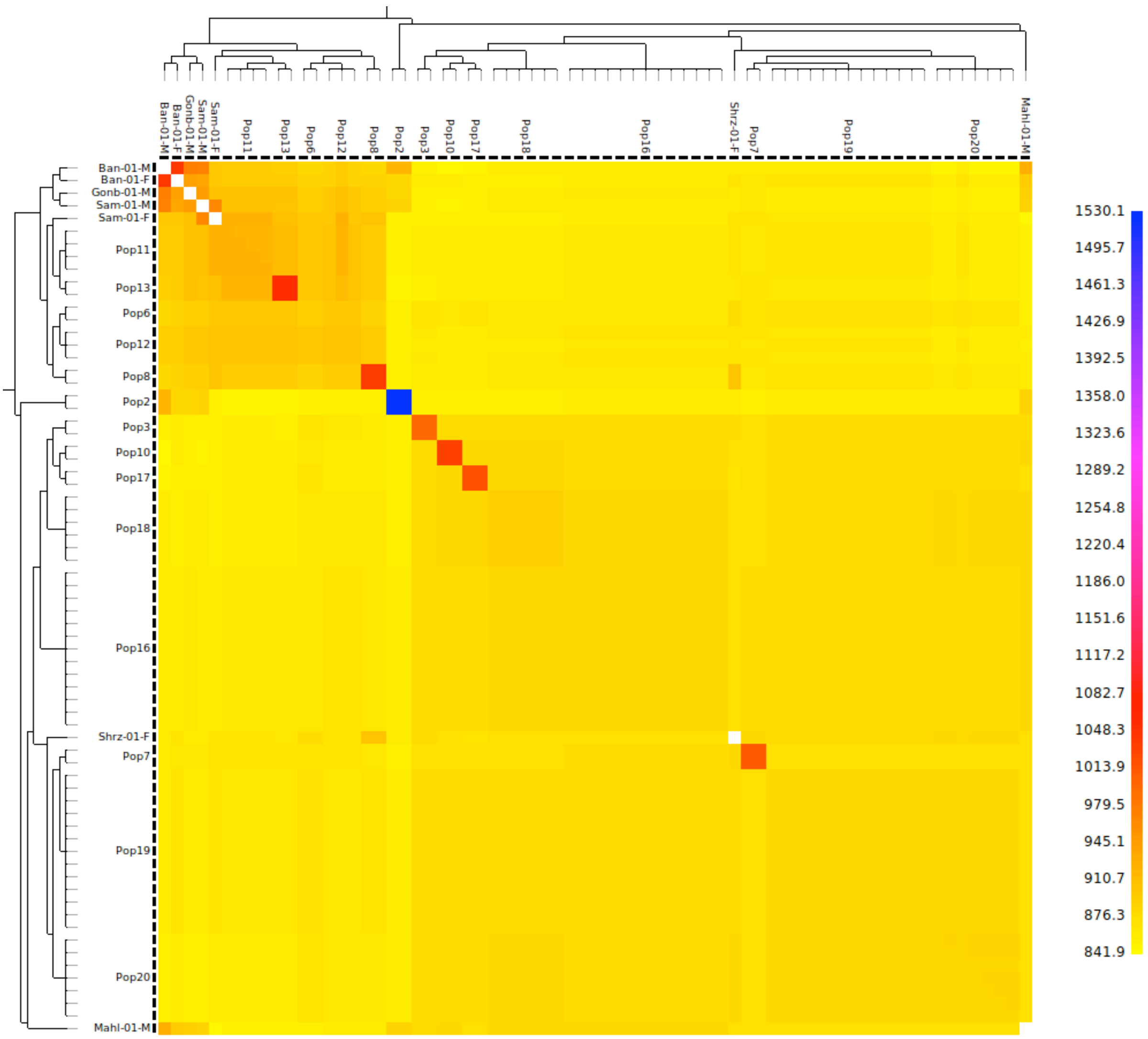

Figure S3: The fineSTRUCTURE clustering with aggregated coancestry matrix for 69 Iranian samples

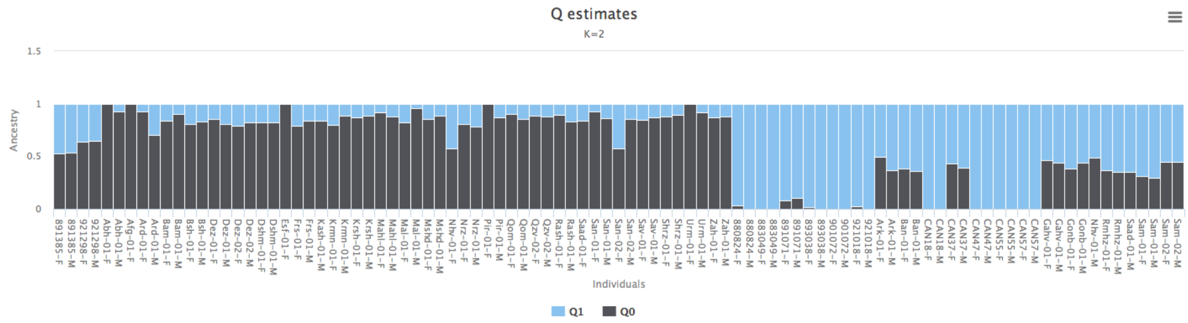

Figure S4: Population structure of Iranian collections (95 samples) estimated using the fastSTRUCTURE admixture model at K = 2.

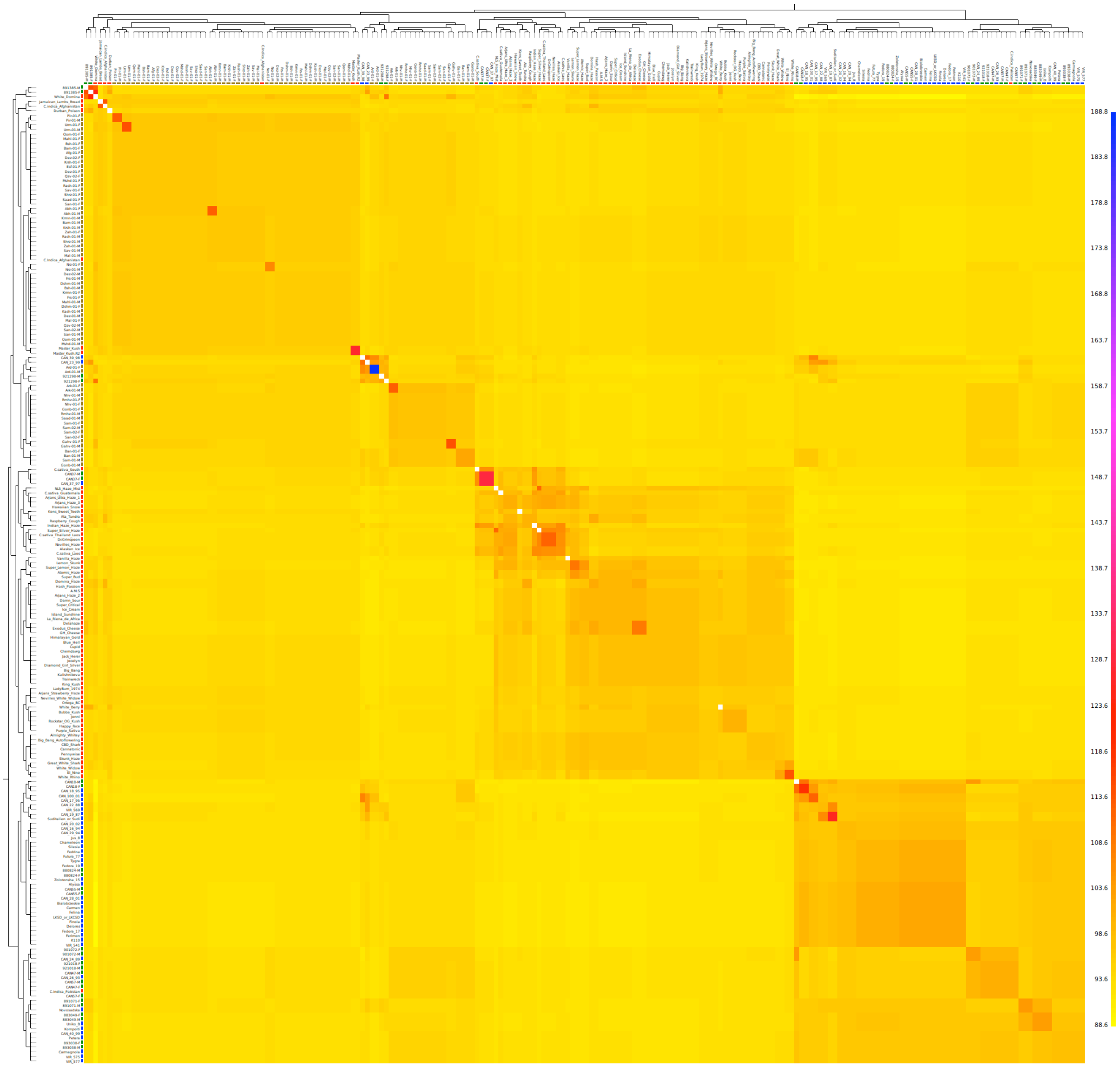

Figure S5: The cluster-level coancestry heat map for 95 samples from Iranian collection, 43 hemp and 71 marijuana samples.
